# Treatment-Free Interval as a Prognostic Factor for Survival Outcomes in Patients with Advanced or Recurrent Endometrial Cancer Treated with Pembrolizumab Plus Lenvatinib: A Multi-Institutional Real-World Cohort Study

**DOI:** 10.3390/cancers18193076

**Published:** 2026-09-22

**Authors:** Chih-Wen Su, Shao-Jing Wang, Yu-Hsiang Shih, Ting-Fang Lu, Yen-Fu Chen, Chun-Ting Fan, Shih-Tien Hsu, Chih-Ku Liu, Lou Sun, Sheau-Feng Hwang, Chien-Hsing Lu, Chia-Che Chang

**Affiliations:** 1 Doctoral Program in Translational Medicine, Rong Hsing Translational Medicine Research Center, National Chung Hsing University, Taichung 402202, Taiwan; zeisler.tw@gmail.com; 2 Department of Anesthesiology, Taichung Veterans General Hospital, Taichung 407219, Taiwan; 3 Department of Gynecology and Obstetrics, Taichung Veterans General Hospital, Taichung 407219, Taiwan; esjdanny@gmail.com (S.-J.W.);; 4 College of Health Care and Management, Chung Shan Medical University, Taichung 402367, Taiwan; 5 Palliative Care Unit, Taichung Veterans General Hospital, Taichung 407219, Taiwan; 6 Center for General Education, Ling Tung University, Taichung 408284, Taiwan; 7 School of Medicine, China Medical University, Taichung 406040, Taiwan; 8 Department of Animal Science and Biotechnology, Tunghai University, Taichung 407224, Taiwan; 9 Department of Food and Nutrition, Providence University, Taichung 433303, Taiwan; 10 Department of Post-Baccalaureate Medicine, College of Medicine, National Chung Hsing University, Taichung 402202, Taiwan; 11 Department of Life Sciences, National Chung Hsing University, Taichung 402202, Taiwan; 12 Graduate Institute of Biomedical Sciences, Rong Hsing Translational Medicine Research Center, National Chung Hsing University, Taichung 402202, Taiwan; 13 The iEGG and Animal Biotechnology Research Center, National Chung Hsing University, Taichung 402202, Taiwan; 14 Department of Medical Laboratory Science and Biotechnology, Asia University, Taichung 413305, Taiwan; 15 Department of Medical Research, China Medical University Hospital, Taichung 406040, Taiwan; 16 Traditional Herbal Medicine Research Center, Taipei Medical University Hospital, Taipei 110301, Taiwan

**Keywords:** endometrial cancer, treatment-free interval, pembrolizumab, lenvatinib, overall survival, real-world evidence

## Abstract

**Background/Objectives:** The treatment-free interval (TFI) predicts benefit from platinum rechallenge in gynecological cancers, but its prognostic value in patients receiving second-line pembrolizumab plus lenvatinib for recurrent endometrial cancer (EC) is unknown. We evaluated whether the TFI is associated with survival and toxicity in this setting. **Methods:** Using the TriNetX Research Network (122 healthcare organizations, United States and Europe), we identified adults aged 18–90 years with advanced or recurrent EC between January 2018 and June 2026 who received three to six cycles of first-line carboplatin plus paclitaxel, progressed, and then received pembrolizumab plus lenvatinib. Patients were stratified by TFI (<6 months, Cohort 1; ≥6 months, Cohort 2) and matched 1:1 by propensity score. The primary outcome was overall survival (OS), compared using Kaplan–Meier and Cox proportional-hazards methods; adverse-event risks were exploratory outcomes compared using *z*-tests for the risk difference, without adjustment for multiple comparisons. **Results:** Of 420 eligible patients, 119 remained in each cohort after matching, with balanced baseline characteristics. Median OS was 10.0 months in Cohort 1 versus 23.6 months in Cohort 2 (hazard ratio 1.60, 95% confidence interval 1.12–2.29; *p* = 0.009), and 3-year OS was 32.5% versus 40.5%. Grade 3 anemia occurred earlier and more often in Cohort 1 (hazard ratio 1.74, 95% confidence interval 1.10–2.76; *p* = 0.017), whereas hypothyroidism was less frequent (31.9% versus 47.1%; *p* = 0.017); the remaining adverse events did not differ significantly between cohorts. **Conclusions:** A TFI ≥ 6 months is associated with significantly longer OS and a largely similar toxicity profile. The TFI is a readily available, real-world evidence-based reference for the prognosis and for the comparative adverse-event profile of the two TFI groups in this setting.

## Simple Summary

Endometrial cancer that returns or spreads after first-line chemotherapy is difficult to treat. The combination of pembrolizumab (an immunotherapy) and lenvatinib (a targeted therapy) is a standard second-line option, but clinicians have few tools for estimating the prognosis of individual patients. Using de-identified electronic health records from 122 healthcare organizations in the United States and Europe, we compared propensity score-matched patients who started pembrolizumab plus lenvatinib within 6 months of their last dose of carboplatin plus paclitaxel with those who started it 6 months or later. This treatment-free interval is measured from the last chemotherapy dose to the start of the next treatment, not from the date the cancer was found to have progressed. Patients with a treatment-free interval of at least 6 months lived significantly longer (median 23.6 versus 10.0 months), whereas most side-effect rates were similar in the two groups; severe anemia was somewhat more common, and hypothyroidism less common, in patients with a short treatment-free interval. The treatment-free interval, which is available for every patient at no additional cost, can help clinicians counsel patients about the survival and the adverse-event profile observed in each treatment-free interval group. Because all patients received the same treatment, this difference most likely reflects the more aggressive biology of tumors that recur quickly, not a difference in how well the treatment works or any advantage of delaying it.

## 1. Introduction

Endometrial cancer (EC) is the sixth most commonly diagnosed malignancy in women, with approximately 420,000 new cases each year worldwide and the highest incidence rates in North America and Europe [1]. The global incidence has risen substantially since 2008, a trend attributed to increasing life expectancy and the growing prevalence of obesity [2]. In the United States, EC is the most prevalent gynecological cancer, and both incidence and mortality are increasing; an estimated 66,200 new cases and 13,030 deaths occurred in 2023 [3]. Whereas the 5-year relative survival rate approaches 95% for localized disease, it falls to approximately 18% in the presence of distant metastases [1,3].

Most patients present with stage I disease and are treated with hysterectomy and bilateral salpingo-oophorectomy with or without nodal assessment [4,5]. Approximately 15% are diagnosed at an advanced stage, for which the 5-year overall survival (OS) rate is 40–65% for International Federation of Gynecology and Obstetrics (FIGO) stage III and 15–17% for stage IVA–B [2]. Cytotoxic chemotherapy—principally platinum salts, taxanes, and anthracyclines—remains the backbone of systemic treatment, and carboplatin plus paclitaxel is the standard first-line regimen for advanced, recurrent, or metastatic disease, with response rates of approximately 30–40% [6,7].

For patients who progress after platinum-based chemotherapy, options are limited. Pembrolizumab monotherapy produces an objective response rate (ORR) of approximately 48% in mismatch repair-deficient (MMRd) or microsatellite instability-high (MSI-H) tumors [8], whereas single-agent lenvatinib, a multitargeted tyrosine kinase inhibitor [9], achieved an ORR of 14.3% in recurrent EC [10]. On the basis of the phase 3 KEYNOTE-775/Study 309 trial, the combination of pembrolizumab and lenvatinib was approved in July 2021 for advanced EC with mismatch repair-proficient tumors that progressed after prior systemic therapy and are not amenable to curative surgery or radiotherapy [11,12]. The combination has since been evaluated in the first-line setting in the ENGOT-en9/LEAP-001 trial [13].

The treatment-free interval (TFI) is an established predictor of benefit from platinum rechallenge in ovarian and cervical cancer, and evidence supports its applicability to EC [14,15]. Whether the TFI also carries prognostic information for survival and tolerability in patients receiving pembrolizumab plus lenvatinib—a regimen that does not contain platinum—has not been established. We therefore conducted a multi-institutional real-world cohort study to address this question.

## 2. Materials and Methods

### 2.1. Data Source

This study used data from the TriNetX Research Network, a federated network of de-identified, Health Insurance Portability and Accountability Act (HIPAA)-compliant, real-time health records aggregated from the electronic health records (EHRs) and cancer registries of participating healthcare organizations (HCOs). At the time of analysis, the network contained data on approximately 250 million patients from 122 HCOs worldwide (as of September 2026). The platform captures diagnoses (ICD-10-CM), procedures (ICD-10-PCS, Current Procedural Terminology [CPT], and HCPCS), medications (RxNorm and the Veterans Affairs National Formulary), laboratory values (LOINC), and genomic information. The study analyzed only de-identified records and did not collect, use, or transmit individually identifiable information. The study protocol was approved by the Institutional Review Board of Taichung Veterans General Hospital (approval no. CE26050A), and the requirement for informed consent was waived. Data were analyzed in September 2026 and covered the study period from 1 January 2018 through 30 June 2026.

### 2.2. Study Design

We conducted a retrospective cohort study to evaluate whether the treatment-free interval between completion of first-line carboplatin plus paclitaxel and initiation of pembrolizumab plus lenvatinib is prognostic for survival and treatment-related adverse events in women with advanced or recurrent endometrial cancer. The study is reported in accordance with the Strengthening the Reporting of Observational Studies in Epidemiology (STROBE) statement.

### 2.3. Eligibility Criteria (Applied to Both Cohorts)

Female patients aged 18 to 90 years with a diagnosis of advanced or recurrent (stage IV) endometrial cancer recorded on or after 1 January 2018 were eligible. Advanced or recurrent disease was operationalized as malignant neoplasm of the endometrium (ICD-10-CM C54.1) together with evidence of secondary or metastatic disease (any of C77.0, C77.1, C77.3, C77.4, C77.8, C77.9, C77, C78, or C79). Patients with carcinosarcoma or sarcoma histology (C54.1 with a histology/behavior of carcinosarcoma, NOS or sarcoma, NOS) were excluded. Patients with any recorded malignant neoplasm of the endometrium between 1 January 1900 and 31 December 2017 were also excluded, to restrict the analysis to incident cases within the study period.

This exclusion was pre-specified to align the study population with the eligibility criteria of the pivotal trials that define the regimen under investigation. Study 309/KEYNOTE-775, which established pembrolizumab plus lenvatinib after prior platinum-based therapy, enrolled women with advanced, recurrent, or metastatic endometrial cancer of any histologic subtype with the exception of carcinosarcoma and sarcoma [12], and the first-line NRG-GY018/KEYNOTE-868 trial of pembrolizumab plus carboplatin and paclitaxel likewise restricted enrolment to histologic subtypes other than carcinosarcoma [16]. Consistent with these criteria, the National Comprehensive Cancer Network (NCCN) Guidelines for Uterine Neoplasms annotate the carboplatin/paclitaxel/pembrolizumab regimen as applicable to stage III–IV disease other than carcinosarcoma, whereas the carboplatin/paclitaxel/dostarlimab regimen, derived from the RUBY trial, carries no such histologic restriction [17,18]. Uterine carcinosarcoma and pure uterine sarcomas are biologically and clinically distinct from endometrial carcinoma, with different patterns of chemosensitivity and a substantially poorer prognosis; retaining them would therefore have introduced a subgroup for which the treatment sequence under study is neither label-supported nor guideline-endorsed, and in which the prognostic meaning of the treatment-free interval has not been established.

Both cohorts were required to have received first-line carboplatin plus paclitaxel, defined as carboplatin (RxNorm 40048 or HCPCS J9045) and paclitaxel (RxNorm 56946 or HCPCS J9267), with between 3 and 6 recorded administrations, followed by pembrolizumab plus lenvatinib, defined as pembrolizumab (RxNorm 1547545 or HCPCS J9271) and lenvatinib (RxNorm 1603296).

### 2.4. Cohort Assignment

The cohorts differed only in the interval between first-line chemotherapy and initiation of pembrolizumab plus lenvatinib. Cohort 1 comprised 198 patients before matching, who began pembrolizumab plus lenvatinib between 1 day and 6 months after first-line carboplatin/paclitaxel; Cohort 2 comprised 222 patients before matching, who began at least 6 months after first-line carboplatin/paclitaxel. The TFI was therefore defined as the interval from the most recent first-line carboplatin/paclitaxel administration to the first administration of pembrolizumab plus lenvatinib; it was not measured from the date of documented progression, since a delay may occur between progression and the start of the next line of therapy. Both cohorts were drawn from the same 122 HCOs queried.

### 2.5. Index Event and Observation Window

The index event for each patient was the first recorded administration of pembrolizumab or lenvatinib. The temporal sequence was therefore: completion of first-line carboplatin plus paclitaxel; documented progression at some point thereafter; and initiation of pembrolizumab plus lenvatinib, which defined both the end of the TFI and the index date. Cohort assignment depended only on the length of the TFI, which was fully determined at the index date, and outcomes were ascertained within a time window beginning 1 day after the index event and ending 1095 days (3 years) after the index event. Because follow-up started at the index date in both cohorts, the interval between the last chemotherapy dose and the index date, during which patients in the longer-TFI cohort necessarily survived and remained eligible, was not counted as follow-up time and therefore did not contribute immortal time to the survival comparison. Index events were required to have occurred within the preceding 20 years; no patients in either cohort were excluded on this basis.

### 2.6. Outcomes

Twenty outcomes were pre-specified. The primary outcome was all-cause mortality. The secondary outcome was a composite of death or sepsis. The remaining 18 outcomes were exploratory treatment-related adverse events, comprising all anemia and grade 3 anemia, all neutropenia and grade 3 neutropenia, elevated liver enzymes, hypertension and grade 3 hypertension, diarrhea, proteinuria, fatigue, abnormal weight loss, arthralgia, nausea and vomiting, hypothyroidism, thrombocytopenia and grade 3 thrombocytopenia, sepsis, and febrile neutropenia. The adverse-event analyses were intended to characterize the safety profile of the regimen in each TFI stratum and to generate hypotheses; they were not powered or prespecified to test individual differences.

Outcomes were defined by diagnosis codes, laboratory thresholds, or both. Laboratory-based severity thresholds were as follows: anemia, hemoglobin < 12.0 g/dL; grade 3 anemia, hemoglobin ≤ 8.0 g/dL; grade 3 neutropenia, absolute neutrophil count ≤ 1.0 × 10^3^/µL; grade 3 thrombocytopenia, platelet count ≤ 50 × 10^3^/µL; grade 3 hypertension, systolic blood pressure ≥ 160 mmHg or diastolic blood pressure ≥ 100 mmHg; and elevated liver enzymes, alanine aminotransferase or aspartate aminotransferase ≥ 50 U/L. Where an outcome definition comprised more than one term, at least one term had to match. Only the most recent laboratory value within the time window was used.

For all 20 outcomes, patients with the outcome recorded before the start of the time window were retained in the analysis. The denominator for every outcome is therefore the full propensity score-matched cohort of 119 patients per group, which permits direct comparison of risks across outcomes, and the matched structure is preserved throughout. As a consequence, the reported cumulative risks combine prevalent conditions recorded before the index date with events first recorded after it, and they should be read as the burden of each condition observed in each cohort during follow-up rather than as the incidence of treatment-emergent events. To address this, the baseline prevalence of each condition (patients with the outcome recorded before day 1) was tabulated, and a sensitivity analysis was performed in which, for each outcome separately, patients with that outcome recorded before the index date were excluded so that only treatment-emergent events were analyzed. Because each outcome excludes a different number of patients from each cohort, the denominators in this sensitivity analysis differ by outcome and the matched pairs are no longer intact. Outcomes for which the patient count in either cohort fell below the data-network reporting threshold (10 patients) are reported as suppressed.

### 2.7. Statistical Analysis

To reduce confounding by measured baseline differences, the two cohorts were matched 1:1 using propensity scores derived from 77 pre-specified baseline covariates spanning demographics (including age at index), comorbid diagnoses, oncology characteristics, procedures (including radiation oncology treatment and chemotherapy lines 1 and 2 [VA class]), medications, and laboratory values. Covariates were selected to capture, as far as the platform allows, the known prognostic determinants of survival in advanced endometrial cancer: age, race and ethnicity; sites of metastatic disease (as ICD-10-CM secondary-neoplasm codes); comorbid diagnoses; recorded tumor biomarkers; prior surgery and radiotherapy; prior lines of systemic therapy and individual antineoplastic agents; and baseline laboratory values including hemoglobin, hematocrit, transaminases, creatinine, and body mass index. Several clinically important prognostic variables—FIGO stage, quantitative metastatic burden, histologic subtype beyond the exclusion of carcinosarcoma and sarcoma, molecular subtype, and performance status—are not reliably available as structured data in the TriNetX platform and could not be included; calendar year of treatment likewise cannot be entered as a matching covariate or used to stratify the analysis, because the platform does not release per-year patient counts. Matching used a greedy nearest-neighbor algorithm without replacement, as implemented in the TriNetX Analytics platform. Covariate balance was assessed using the standardized difference, with an absolute value below 0.1 taken to indicate adequate balance.

For each outcome, the risk was calculated as the number of patients with the outcome divided by the number of patients in the cohort. Between-cohort comparisons were expressed as the risk difference, risk ratio, and odds ratio, each with a 95% confidence interval; the risk difference was tested using a *z*-test.

Time-to-event outcomes were analyzed using the Kaplan–Meier method with daily intervals. Patients were censored at the date of the last recorded fact in their EHR. We report the median time to event (defined as the number of days at which the event-free probability falls below 50%; “not reached” where this did not occur within the window), the event-free probability at the end of the 1095-day window, the log-rank test, and the hazard ratio with its 95% confidence interval. The proportional hazards assumption was formally tested for each outcome; where it was violated, the hazard ratio is reported as a time-averaged effect and interpreted alongside the median event-free times. The hazard ratios obtained for the 20 outcomes were additionally summarized together in a forest plot to allow the direction and precision of every comparison to be inspected side by side. As a sensitivity analysis, the entire procedure (cohort construction, 1:1 propensity score matching on the same 77 covariates, and Kaplan–Meier analysis from the same index date) was repeated with the TFI dichotomized at 3, 9, and 12 months instead of 6 months, to examine whether the associations depended on the threshold chosen and to address the possibility that cohort definition by a future interval had introduced selection bias.

All analyses were performed within the TriNetX Analytics platform (TriNetX LLC, Cambridge, MA, USA), which executes its statistical routines in R version 4.0.2 (R Foundation for Statistical Computing, Vienna, Austria) using the survival package for Kaplan–Meier estimation, log-rank testing, and Cox proportional-hazards modeling. Propensity score matching was performed with the platform’s built-in greedy nearest-neighbor implementation. A two-sided *p* value below 0.05 was considered statistically significant. No adjustment for multiple comparisons was applied. The TriNetX Analytics platform executes each outcome analysis separately and returns only aggregate results, without access to patient-level data, so a formal multiplicity-adjusted analysis that accounts for the correlation among outcomes could not be implemented. Because the primary outcome was a single prespecified comparison, its *p* value is reported unadjusted. The results for the 17 reportable exploratory outcomes should therefore be interpreted with caution, in light of the increased risk of type I error.

## 3. Results

### 3.1. Cohort Selection and Baseline Characteristics

Between 1 January 2018 and 30 June 2026, adults aged 18 to 90 years with advanced or recurrent endometrial cancer were identified across the 122 participating healthcare organizations. Of these, 420 had received between 3 and 6 administrations of first-line carboplatin plus paclitaxel, progressed, and subsequently received pembrolizumab plus lenvatinib, and were therefore eligible for analysis. A total of 198 patients met the criteria for Cohort 1 (TFI < 6 months) and 222 patients met the criteria for Cohort 2 (TFI ≥ 6 months). After 1:1 propensity score matching on the 77 pre-specified covariates, 119 patients remained in each cohort (Figure 1).

After matching, all 77 covariates achieved a standardized difference below 0.1, and the cohorts were well balanced on demographic and clinical characteristics (Table 1). Mean age at index was 67.5 ± 9.0 years in Cohort 1 and 67.1 ± 9.0 years in Cohort 2; 66.4% and 58.8% of patients, respectively, were White and 23.5% and 26.1% were Black or African American. The distribution of metastatic sites was similar, with secondary neoplasm of the respiratory and digestive organs recorded in 52.9% versus 55.5%, other and unspecified sites in 48.7% versus 52.1%, lymph nodes in 46.2% versus 45.4%, and retroperitoneum or peritoneum in 31.9% versus 33.6%. Prior surgery on the female genital system (74.8% versus 75.6%), radiation oncology treatment (37.0% versus 38.7%), and line-3 chemotherapy (37.8% versus 38.7%) were likewise comparable, and all patients in both cohorts had received carboplatin, paclitaxel, and lenvatinib. Body mass index was similar (30.3 versus 30.1 kg/m^2^). Baseline hemoglobin (10.6 versus 11.5 g/dL) and hematocrit (32.4% versus 35.3%) nevertheless remained lower in Cohort 1 after matching, a residual imbalance in the direction of greater baseline anemia in the shorter-interval group that is relevant to the interpretation of the anemia outcomes.

**Table 1:** Baseline characteristics after propensity score matching.

Characteristic	Cohort 1 (TFI < 6 Months), *n* = 119	Cohort 2 (TFI ≥ 6 Months), *n* = 119
**Demographics**		
Age at index, years, mean ± SD	67.5 ± 9.0	67.1 ± 9.0
Current age, years, mean ± SD	71.1 ± 8.9	70.6 ± 9.1
White	79 (66.4)	70 (58.8)
Black or African American	28 (23.5)	31 (26.1)
Asian	≤10 (≤8.4)	≤10 (≤8.4)
Not Hispanic or Latino	105 (88.2)	99 (83.2)
Hispanic or Latino	≤10 (≤8.4)	≤10 (≤8.4)
Unknown ethnicity	11 (9.2)	15 (12.6)
**Sites of metastatic disease**		
Ill-defined, other secondary and unspecified sites	107 (89.9)	108 (90.8)
Respiratory and digestive organs	63 (52.9)	66 (55.5)
Other and unspecified sites	58 (48.7)	62 (52.1)
Lymph nodes, secondary and unspecified	55 (46.2)	54 (45.4)
Retroperitoneum and peritoneum	38 (31.9)	40 (33.6)
Intrapelvic lymph nodes	34 (28.6)	34 (28.6)
Lung	21 (17.6)	22 (18.5)
Liver and intrahepatic bile duct	16 (13.4)	16 (13.4)
Intra-abdominal lymph nodes	16 (13.4)	16 (13.4)
Large intestine and rectum	≤10 (≤8.4)	≤10 (≤8.4)
**Biomarkers recorded ^a^**		
HER2	67 (56.3)	69 (58.0)
Estrogen receptor	66 (55.5)	68 (57.1)
Progesterone receptor	62 (52.1)	66 (55.5)
Microsatellite instability	≤10 (≤8.4)	≤10 (≤8.4)
KRAS	≤10 (≤8.4)	≤10 (≤8.4)
Ki-67	≤10 (≤8.4)	≤10 (≤8.4)
**Prior procedures**		
Surgery on the female genital system	89 (74.8)	90 (75.6)
Surgery on the corpus uteri	75 (63.0)	73 (61.3)
Radiation oncology treatment	44 (37.0)	46 (38.7)
**Systemic therapy**		
Carboplatin	119 (100)	119 (100)
Paclitaxel	119 (100)	119 (100)
Lenvatinib	119 (100)	119 (100)
Chemotherapy, line 1	119 (100)	119 (100)
Chemotherapy, line 2	119 (100)	119 (100)
Chemotherapy, line 3	45 (37.8)	46 (38.7)
Bevacizumab	≤10 (≤8.4)	≤10 (≤8.4)
Doxorubicin	≤10 (≤8.4)	≤10 (≤8.4)
Docetaxel	≤10 (≤8.4)	≤10 (≤8.4)
**Laboratory values, mean ± SD**		
Hemoglobin, g/dL	10.6 ± 1.64	11.5 ± 1.71
Hematocrit, %	32.4 ± 5.58	35.3 ± 4.85
Alanine aminotransferase, U/L	17.1 ± 9.66	18.8 ± 11.3
Aspartate aminotransferase, U/L	23.1 ± 14.1	23.4 ± 12.2
Creatinine, mg/dL	0.97 ± 0.74	0.90 ± 0.28
Body mass index, kg/m^2^	30.3 ± 7.83	30.1 ± 7.54
Height, inches	63.5 ± 2.90	63.5 ± 2.78

SD, standard deviation. Cohort 1 = treatment-free interval < 6 months; Cohort 2 = treatment-free interval ≥ 6 months. Covariates were measured in the window ending on the day of the index event (first administration of pembrolizumab plus lenvatinib). Values are *n* (%) unless otherwise indicated. Cohorts were matched 1:1 by propensity score on 77 baseline covariates; 198 and 222 eligible patients yielded 119 matched pairs. All 77 covariates achieved a standardized difference below 0.1 after matching; the table shows a prespecified subset, and the full covariate list is available on request. ^a^ Entries indicate that the biomarker was recorded in the record, not that the result was positive; the platform does not release the result value for most of these assays. Counts of 10 or fewer are displayed by the data network as “10” and are shown here as ≤10; several rows sit at this threshold in both cohorts, so the apparent perfect equality in those rows reflects censoring of small counts rather than exact balance and should not be read as evidence of successful matching. Residual imbalance: hemoglobin (10.6 versus 11.5 g/dL) and hematocrit (32.4% versus 35.3%) remain lower in Cohort 1 after matching, both in the direction of greater anemia in the shorter-interval group; this is directly relevant to the anemia outcomes in Table 2 and Table 3. Laboratory values were available for 112 to 119 patients per cohort except for carcinoembryonic antigen and cancer antigen 19-9, which were recorded in fewer than 16 patients per cohort and are omitted. Missing laboratory data are unlikely to be missing at random, since testing is ordered in response to clinical suspicion.

**Table 2:** Measures of association for all outcomes, propensity score-matched cohorts.

Outcome	Patients with Outcome		Risk, %		Risk Difference			Risk Ratio	Odds Ratio
	Cohort 1	Cohort 2	Cohort 1	Cohort 2	% (95% CI)	*z*	*p*		
**Mortality**									
Death ^a^	67	55	56.3	46.2	10.08 (−2.55 to 22.72)	1.564	0.120	1.218	1.499
Death or sepsis	75	64	63.0	53.8	9.24 (−3.23 to 21.71)	1.447	0.148	1.172	1.465
**Hematologic toxicity**									
Anemia, any	105	103	88.2	86.6	1.68 (−6.75 to 10.11)	0.391	0.696	1.019	1.165
Anemia, grade 3 ^b^	44	31	37.0	26.1	10.92 (−0.80 to 22.65)	1.813	0.070	1.419	1.665
Neutropenia, any	16	15	13.4	12.6	0.84 (−7.71 to 9.39)	0.192	0.847	1.067	1.077
Neutropenia, grade 3 ^c^	*Suppressed—patient count below the data-network reporting threshold*								
Thrombocytopenia, any	15	15	12.6	12.6	0.00 (−8.43 to 8.43)	0.000	1.000	1.000	1.000
Thrombocytopenia, grade 3 ^d^	*Suppressed—patient count below the data-network reporting threshold*								
**Lenvatinib-associated toxicity**									
Hypertension	80	82	67.2	68.9	−1.68 (−13.53 to 10.16)	−0.278	0.781	0.976	0.926
Hypertension, grade 3 ^e^	69	75	58.0	63.0	−5.04 (−17.45 to 7.36)	−0.796	0.426	0.920	0.810
Proteinuria	21	25	17.6	21.0	−3.36 (−13.39 to 6.66)	−0.657	0.511	0.840	0.806
Elevated liver enzymes ^f^	52	57	43.7	47.9	−4.20 (−16.85 to 8.45)	−0.651	0.515	0.912	0.844
Diarrhea	34	37	28.6	31.1	−2.52 (−14.14 to 9.10)	−0.425	0.671	0.919	0.886
**Immune-related and constitutional**									
Hypothyroidism	38	56	31.9	47.1	**−15.13 (−27.40 to −2.85)**	−2.387	**0.017**	0.679	0.528
Arthralgia	28	35	23.5	29.4	−5.88 (−17.07 to 5.30)	−1.029	0.304	0.800	0.738
Fatigue	44	42	37.0	35.3	1.68 (−10.52 to 13.89)	0.270	0.787	1.048	1.076
Nausea and vomiting	60	52	50.4	43.7	6.72 (−5.93 to 19.38)	1.041	0.299	1.154	1.310
Weight loss	17	15	14.3	12.6	1.68 (−6.99 to 10.35)	0.380	0.704	1.133	1.156
**Infection**									
Sepsis	17	19	14.3	16.0	−1.68 (−10.78 to 7.42)	−0.362	0.718	0.895	0.877
Febrile neutropenia ^g^	*Suppressed—patient count below the data-network reporting threshold*								

CI, confidence interval. Cohort 1 = treatment-free interval < 6 months (*n* = 119); Cohort 2 = treatment-free interval ≥ 6 months (*n* = 119). Patients with the outcome recorded before the time window were included, and outcomes were ascertained from day 1 to day 1095 after the first administration of pembrolizumab plus lenvatinib. Cohorts were matched 1:1 by propensity score on 77 baseline covariates; 198 and 222 eligible patients yielded 119 matched pairs, and the full 119 pairs contributed to every outcome. Risk difference, risk ratio, and odds ratio are for Cohort 1 versus Cohort 2, so a risk difference above zero indicates that the outcome was more common in patients with a treatment-free interval shorter than 6 months; the risk difference was tested by *z*-test. Bold denotes *p* < 0.05. ^a^ One patient in Cohort 1 was not evaluable for mortality. ^b^ Hemoglobin ≤ 8 g/dL or ICD-10-CM D64. ^c^ Absolute neutrophil count ≤ 1 × 10^3^/µL. ^d^ Platelet count ≤ 50 × 10^3^/µL. ^e^ Systolic blood pressure ≥ 160 mmHg or diastolic blood pressure ≥ 100 mmHg. ^f^ Alanine aminotransferase or aspartate aminotransferase ≥ 50 U/L. ^g^ ICD-10-CM R50.81. Hypothyroidism is the only outcome with a significant risk difference, and its direction is opposite to that of the mortality and anemia signals; the corresponding hazard ratio in Table 3 (0.668, 95% CI 0.442–1.008) does not reach significance, so this finding rests on cumulative incidence rather than on time to event and should be interpreted cautiously. Any anemia occurred in 86% to 88% of patients in both cohorts, so this endpoint has little discriminatory value; the grade 3 threshold is the informative one, and hemoglobin and hematocrit remained imbalanced after matching (Table 1), which bears directly on both anemia endpoints. Three of 20 prespecified outcomes could not be reported because the patient count fell below the data-network reporting threshold. No adjustment for multiple comparisons was applied.

**Table 3:** Kaplan–Meier survival analysis for all outcomes, propensity score-matched cohorts.

Outcome	No. with Outcome		Median Event-Free Time, d		Event-Free Probability at 1095 d, %		Log-Rank Test		Hazard Ratio (95% CI)	Proportionality *p*
	C1	C2	C1	C2	C1	C2	χ^2^	*p*		
**Mortality**										
Death ^a^	67	55	305	719	32.5	40.5	6.786	**0.009**	**1.600 (1.119–2.288)**	**0.014**
Death or sepsis	75	64	238	513	25.2	34.5	8.058	**0.005**	**1.617 (1.156–2.262)**	**0.033**
**Hematologic toxicity**										
Anemia, any	105	103	21	22	4.1	7.0	3.093	0.079	1.274 (0.970–1.675)	0.301
Anemia, grade 3 ^b^	44	31	NR	NR	51.5	64.9	5.664	**0.017**	**1.738 (1.096–2.756)**	0.146
Neutropenia, any	16	15	NR	NR	83.8	81.0	0.244	0.622	1.195 (0.590–2.419)	**0.035**
Neutropenia, grade 3 ^c^	*Suppressed—patient count below the data-network reporting threshold*									
Thrombocytopenia, any	15	15	NR	NR	81.9	82.4	0.139	0.709	1.146 (0.560–2.346)	0.246
Thrombocytopenia, grade 3 ^d^	*Suppressed—patient count below the data-network reporting threshold*									
**Lenvatinib-associated toxicity**										
Hypertension	80	82	74	67	20.8	21.9	0.008	0.931	1.013 (0.744–1.378)	0.285
Hypertension, grade 3 ^e^	69	75	94	123	25.1	21.9	0.055	0.815	0.959 (0.691–1.331)	0.685
Proteinuria	21	25	NR	NR	79.9	73.6	0.254	0.615	0.862 (0.482–1.540)	0.467
Elevated liver enzymes ^f^	52	57	597	436	42.3	40.1	0.001	0.980	1.005 (0.690–1.464)	0.142
Diarrhea	34	37	NR	NR	60.0	57.2	0.009	0.925	1.023 (0.642–1.631)	0.288
**Immune-related and constitutional**										
Hypothyroidism	38	56	NR	289	55.9	41.7	3.740	0.053	0.668 (0.442–1.008)	0.874
Arthralgia	28	35	NR	NR	64.5	54.2	0.080	0.778	0.931 (0.566–1.532)	0.293
Fatigue	44	42	NR	NR	50.4	53.4	0.709	0.400	1.200 (0.786–1.834)	0.343
Nausea and vomiting	60	52	281	630	34.6	43.9	2.221	0.136	1.325 (0.913–1.922)	0.413
Weight loss	17	15	NR	NR	79.5	82.5	0.515	0.473	1.290 (0.643–2.586)	0.484
**Infection**										
Sepsis	17	19	NR	NR	79.6	77.2	0.027	0.870	1.056 (0.548–2.036)	0.320
Febrile neutropenia ^g^	*Suppressed—patient count below the data-network reporting threshold*									

C1, Cohort 1 (treatment-free interval < 6 months); C2, Cohort 2 (treatment-free interval ≥ 6 months); CI, confidence interval; d, days; NR, not reached. Patients with the outcome recorded before the time window were included; follow-up from day 1 to day 1095 after the first administration of pembrolizumab plus lenvatinib. Cohorts were matched 1:1 by propensity score on 77 baseline covariates; 198 and 222 eligible patients yielded 119 matched pairs, and the full 119 pairs contributed to every outcome. “Median event-free time” is the time at which half the cohort had experienced the outcome; NR indicates that fewer than half the patients experienced the outcome within 1095 days. Hazard ratios are for Cohort 1 versus Cohort 2, so a value above 1 indicates that the outcome occurred earlier in patients with a treatment-free interval shorter than 6 months. Patients were censored at the last recorded follow-up. Bold denotes a 95% CI excluding the null or *p* < 0.05. Footnotes ^a–g^ as in Table 2. The proportional-hazards assumption was violated for death (*p* = 0.014), death or sepsis (*p* = 0.033), and any neutropenia (*p* = 0.035); for the two mortality endpoints the hazard ratio is therefore a time-averaged effect. The divergence in median event-free time (305 versus 719 days for death) indicates that the excess risk is concentrated in the first year, and a restricted mean survival time or a piecewise model should be reported alongside the hazard ratio. Three of 20 prespecified outcomes could not be reported because the patient count fell below the data-network reporting threshold. No adjustment for multiple comparisons was applied.

### 3.2. Mortality

Patients with a TFI < 6 months (Cohort 1) had numerically higher mortality than those with a TFI ≥ 6 months (Cohort 2). Death occurred in 67 of 119 patients (56.3%) in Cohort 1 compared with 55 of 119 patients (46.2%) in Cohort 2, an absolute risk difference of 10.1 percentage points (95% CI −2.6 to 22.7; *z* = 1.564; *p* = 0.120; risk ratio 1.218; odds ratio 1.499), a difference that did not reach statistical significance in the risk analysis.

Kaplan–Meier analysis, however, showed a markedly shorter median survival in Cohort 1 (305 days versus 719 days) and a lower survival probability at the end of the 3-year window (32.5% versus 40.5%). The difference was statistically significant on the log-rank test (χ^2^ = 6.786, df = 1; *p* = 0.009), with a hazard ratio of 1.600 (95% CI 1.119 to 2.288). The test of proportionality was significant (*p* = 0.014), indicating that the hazard ratio should be interpreted as a time-averaged effect; the curves separated early and ran close to parallel after roughly one year, indicating that the excess risk in Cohort 1 was concentrated in the first year after the index event (Figure 2).

The composite outcome of death or sepsis followed the same pattern, occurring in 75 of 119 patients (63.0%) versus 64 of 119 patients (53.8%) (risk difference 0.092, 95% CI −0.032 to 0.217; *p* = 0.148; odds ratio 1.465, 95% CI 0.873 to 2.459). Median event-free time was 238 days versus 513 days, with 3-year event-free probabilities of 25.2% and 34.5%, respectively (log-rank χ^2^ = 8.058; *p* = 0.005; hazard ratio 1.617, 95% CI 1.156 to 2.262; Figure 3, Table 3). The proportional hazards assumption was again violated (*p* = 0.033), so this hazard ratio likewise represents a time-averaged effect.

### 3.3. Hematologic Adverse Events

The cumulative risk of any anemia was high and similar in both cohorts (88.2% versus 86.6%; risk difference 0.017, 95% CI −0.068 to 0.101; *p* = 0.696). Nearly all patients in both cohorts developed anemia within three weeks of the index event (median time to anemia 21 days versus 22 days; log-rank *p* = 0.079; hazard ratio 1.274, 95% CI 0.970 to 1.675), so this endpoint had little discriminatory value. Grade 3 anemia, in contrast, was more frequent in Cohort 1 (37.0% versus 26.1%; risk difference 0.109, 95% CI −0.008 to 0.227; *p* = 0.070) and occurred significantly earlier in the time-to-event analysis (log-rank *p* = 0.017; hazard ratio 1.738, 95% CI 1.096 to 2.756), with 3-year event-free probabilities of 51.5% versus 64.9%; the median time to grade 3 anemia was not reached in either cohort.

Neutropenia showed no significant between-cohort difference when assessed as any neutropenia (13.4% versus 12.6%; *p* = 0.847; hazard ratio 1.195, 95% CI 0.590 to 2.419), and any thrombocytopenia occurred in 12.6% of both cohorts (*p* = 1.000; hazard ratio 1.146, 95% CI 0.560 to 2.346). Grade 3 neutropenia and grade 3 thrombocytopenia could not be reported because the patient count in at least one cohort fell below the data-network reporting threshold.

### 3.4. Non-Hematologic Adverse Events

None of the lenvatinib-associated adverse events differed significantly between cohorts in either the risk or the survival analysis. Hypertension occurred in 67.2% versus 68.9% (*p* = 0.781), grade 3 hypertension in 58.0% versus 63.0% (*p* = 0.426), proteinuria in 17.6% versus 21.0% (*p* = 0.511), elevated liver enzymes in 43.7% versus 47.9% (*p* = 0.515), and diarrhea in 28.6% versus 31.1% (*p* = 0.671). Among immune-related and constitutional outcomes, hypothyroidism was significantly less frequent in Cohort 1 (31.9% versus 47.1%; risk difference −0.151, 95% CI −0.274 to −0.029; risk ratio 0.679, 95% CI 0.491 to 0.938; odds ratio 0.528, 95% CI 0.311 to 0.894; *p* = 0.017); the corresponding time-to-event analysis was of borderline significance (log-rank *p* = 0.053; hazard ratio 0.668, 95% CI 0.442 to 1.008), with a median time to hypothyroidism of 289 days in Cohort 2 that was not reached in Cohort 1. Arthralgia occurred in 23.5% versus 29.4% (*p* = 0.304), fatigue in 37.0% versus 35.3% (*p* = 0.787), nausea and vomiting in 50.4% versus 43.7% (*p* = 0.299), abnormal weight loss in 14.3% versus 12.6% (*p* = 0.704), and sepsis in 14.3% versus 16.0% (*p* = 0.718). Febrile neutropenia could not be reported because the patient count fell below the reporting threshold. Of the 20 pre-specified outcomes, 17 were reportable; the complete results appear in Table 2 (risk analysis) and Table 3 (Kaplan–Meier analysis), and the hazard ratios for all reportable outcomes are displayed together in Figure 3.

### 3.5. Summary of Findings

After propensity score matching, a TFI < 6 months between first-line carboplatin and paclitaxel and second-line pembrolizumab plus lenvatinib was associated with worse overall survival, a higher combined risk of death or sepsis, and earlier and more frequent grade 3 anemia compared with a TFI ≥ 6 months, whereas hypothyroidism was less frequent in the short-TFI group. The adverse-event comparisons were exploratory and unadjusted for multiplicity, so the two differences observed (grade 3 anemia and hypothyroidism, both *p* = 0.017) should be regarded as hypothesis-generating. The overall adverse-event profile was otherwise similar between the two groups, with no significant differences across the remaining 13 reportable outcomes, whose confidence intervals all crossed a hazard ratio of 1 (Figure 3); three outcomes could not be reported because of small patient counts. Because every patient received the same regimen, these associations describe prognosis within a uniformly treated population; they do not indicate that the timing of pembrolizumab plus lenvatinib modified its effect.

### 3.6. Sensitivity Analysis with Alternative TFI Thresholds

Repeating the analysis with the TFI dichotomized at 3, 9, and 12 months yielded 77, 94, and 65 matched pairs, respectively (Table 4). The direction of every association was unchanged across thresholds. The hazard ratio for death ranged from 1.44 to 1.60 and was largest and statistically significant only at the 6-month threshold (HR 1.60; *p* = 0.009), with *p* values of 0.08–0.10 at the other cut-offs; death or sepsis was significant at 3 and 6 months. Grade 3 anemia was significantly more frequent in the shorter-interval cohort at every threshold (HR 1.74–3.01), any anemia at 3, 9, and 12 months, and hypothyroidism less frequent at 3 months only. The consistency of direction across cut-offs, together with the strongest and most precise mortality signal at 6 months, supports the clinical relevance of the conventional 6-month threshold in this setting and argues against the survival difference being an artifact of the particular cut-off chosen.

### 3.7. Baseline Prevalence and Sensitivity Analysis Restricted to Treatment-Emergent Events

The baseline prevalence of each adverse-event condition and the results of the analysis restricted to patients without the corresponding condition before the index date are shown in Table 5. Prevalent conditions were common: hypertension was already recorded in 69.7% and 68.9% of patients in Cohorts 1 and 2, grade 3 anemia in 47.1% and 31.9%, nausea and vomiting in 41.2% and 46.2%, fatigue in 35.3% of both cohorts, arthralgia in 36.1% and 39.5%, elevated liver enzymes in 31.1% and 33.6%, diarrhea in 23.5% and 20.2%, and hypothyroidism in 14.3% and 20.2%. After exclusion of prevalent cases, seven outcomes (any anemia, any and grade 3 neutropenia, any and grade 3 thrombocytopenia, proteinuria, sepsis, and febrile neutropenia) could no longer be reported because the residual patient count fell below the data-network threshold. For the outcomes that remained reportable, the point estimates were essentially unchanged while the confidence intervals widened with the reduced denominators. The hazard ratio for death was identical (1.601); death or sepsis moved from 1.617 to 1.508 (95% CI 1.060–2.145; *p* = 0.021) and remained significant; grade 3 anemia moved from 1.738 to 1.744 (95% CI 0.859–3.541), with the loss of statistical significance attributable to the reduction in patients at risk from 119 to 63 and 81 rather than to any change in effect size; and the risk ratio for hypothyroidism moved from 0.679 to 0.718 (hazard ratio 0.668 to 0.811), again with a wider interval. The lenvatinib-associated and constitutional outcomes remained non-significant, as in the main analysis. Grade 3 hypertension showed an unstable pattern (median event-free time 966 versus 203 days but identical 3-year event-free probability of 30.4%) that reflects the small numbers at risk (35 and 29) and should not be interpreted. Taken together, the stability of the point estimates indicates that the associations observed in the main analysis are not an artifact of prevalent baseline conditions.

## 4. Discussion

In this real-world analysis of patients with recurrent EC treated across the United States and Europe, the TFI was a significant prognostic determinant of survival after second-line pembrolizumab plus lenvatinib. All patients received the same regimen, so the comparison is between prognostic strata within a uniformly treated population, not between treatment strategies. Patients with a TFI ≥ 6 months survived a median of 23.6 months compared with 10.0 months for those with a TFI < 6 months (HR 1.60; *p* = 0.009), and the difference in 3-year OS (40.5% versus 32.5%) represents a clinically meaningful gain in a population with historically poor outcomes [19,20]. The composite of death or sepsis showed the same pattern and magnitude, reinforcing the consistency of the mortality signal. Because the proportional hazards assumption was not met for either mortality endpoint, the hazard ratios are best read as time-averaged effects; the wide separation of the median survival times (305 versus 719 days) indicates that the excess mortality in the short-TFI group was concentrated in the first year after initiation.

After recurrence, the principal options are platinum rechallenge or an immune checkpoint inhibitor (ICI)-based combination. Alternative second-line cytotoxic regimens generally produce modest response rates and distinct toxicity profiles [6,19]. For patients with a TFI < 6 months, platinum rechallenge is associated with low response rates and appreciable toxicity [21], whereas pembrolizumab plus lenvatinib confers superior OS relative to conventional chemotherapy [12]. The TFI therefore offers a practical marker for estimating the survival that can be expected in patients who receive combined immunotherapy and antiangiogenic therapy [15,22], and may help to frame the discussion of the substantial cost of this regimen in relation to the expected clinical course [23]. The most plausible explanation for this finding is not that earlier treatment is harmful or that deferring treatment is beneficial, but that the TFI functions as a surrogate for platinum sensitivity, disease burden, and tumor aggressiveness. Patients who require a switch to pembrolizumab plus lenvatinib within 6 months of platinum-based chemotherapy are, by definition, those who progressed early; the clinical phenotype is conventionally described as platinum-resistant or platinum-refractory disease. A short platinum-free interval is one of the most robust adverse prognostic markers in gynecologic oncology, established first in ovarian cancer and increasingly recognized in endometrial cancer. Cohort 1 therefore represents a biologically more aggressive population, and the observed survival difference is best interpreted as a prognostic association rather than as evidence that deferring immunotherapy improves outcome. Because no comparator regimen was included, our data cannot address whether pembrolizumab plus lenvatinib is more or less effective in either TFI stratum; that question would require a comparison with an alternative treatment within each stratum, ideally in a randomized design.

This interpretation is supported by the adverse-event data. If the regimen were intrinsically more toxic in patients with a short TFI, one would expect a broad excess of treatment-related events in Cohort 1. Instead, the characteristic toxicity signature of lenvatinib—hypertension, grade 3 hypertension, proteinuria, diarrhea, and elevated liver enzymes—together with fatigue and arthralgia was statistically indistinguishable between cohorts, and 13 of the 17 reportable outcomes showed no significant difference (Figure 3). The excess of grade 3 anemia in Cohort 1 (HR 1.74) requires careful interpretation. What the data show is that baseline hemoglobin was already lower in Cohort 1 after matching (10.6 versus 11.5 g/dL), that the cumulative incidence of any anemia was nearly identical (88.2% versus 86.6%), and that the difference was confined to the grade 3 threshold; these observations establish that the two cohorts entered the observation window with different hematologic reserve, and they are compatible with the excess of grade 3 anemia arising, at least in part, from this baseline difference rather than from a distinct toxicity mechanism. We hypothesize that the lower baseline hemoglobin reflects greater tumor burden and reduced marrow reserve after recent cytotoxic exposure in patients who progressed rapidly; however, tumor burden, marrow function, and the interval since the last cytotoxic dose were not measured directly, and the data available cannot distinguish this explanation from a genuinely greater hematologic toxicity of the regimen in short-TFI disease. This hypothesis would need to be tested in a dataset with patient-level hemoglobin trajectories and transfusion records. Similarly, the lower frequency of hypothyroidism in Cohort 1 (31.9% versus 47.1%) is, in our view, most plausibly explained by the shorter survival and treatment exposure of this group, which allows less time for an immune-related endocrinopathy to emerge, rather than by any effect of the TFI itself on treatment tolerability; this too is a hypothesis, since time on treatment was not available in the dataset, and it should be regarded as consistent with the survival difference rather than as an independent finding.

Notably, despite the difference in survival, adverse-event profiles were broadly comparable between cohorts, indicating that the TFI does not materially influence the tolerability of pembrolizumab plus lenvatinib. Because the between-cohort comparison did not indicate greater toxicity in the short-TFI group, counseling in these patients can focus on prognosis rather than on heightened toxicity concerns [19,24], although closer monitoring of hemoglobin may be warranted in this group given its lower baseline values.

For ICI-naïve patients, ICI monotherapy remains a viable alternative [25,26,27]. KEYNOTE-158 established pembrolizumab as an effective agent in MMRd/MSI-H EC, with an ORR of approximately 48% and durable responses, alongside a lower incidence of grade 3 adverse events than cytotoxic chemotherapy [8,27]. The GARNET trial similarly reported ORRs of 42–45% with dostarlimab in MMRd/MSI-H tumors, with the median duration of response not reached at extended follow-up [28,29]. However, MMRd tumors account for only about a quarter of advanced or recurrent EC [30], which limits the applicability of biomarker-selected ICI monotherapy. In a landscape where most alternatives are either biomarker-restricted or of modest benefit, a prognostic parameter that is available for every patient at no additional cost assumes correspondingly greater practical value, and its biological basis therefore warrants closer examination.

The concordance of the 6-month TFI threshold with observations in ovarian and cervical cancer suggests shared biological mechanisms of treatment resistance [31,32]. Although pembrolizumab plus lenvatinib remains an option across TFI subgroups, survival on the regimen is shorter in patients with a short TFI. Because all patients in the present study received the same regimen, this observation describes prognosis and not a reduced treatment effect; whether the TFI also modifies the benefit of the regimen relative to alternatives remains untested. The prognostic difference probably reflects underlying tumor biology, in which rapid recurrence correlates with intrinsic resistance features such as enhanced DNA-repair capacity, upregulated drug efflux, and specific genomic alterations [33,34]. In addition, chemotherapy-induced remodeling of the tumor immune microenvironment may produce persistent immunosuppression that contributes to the poorer outcomes observed in short-TFI disease despite the antiangiogenic contribution of lenvatinib [11], although this mechanism remains hypothetical in the absence of a comparator arm. Comprehensive molecular profiling will be required to characterize the genomic and immunological drivers of resistance in rapidly progressive disease [33,34,35].

Cancer antigen 125 (CA-125) is most familiar as a tumor marker in ovarian cancer, but it also carries prognostic information in endometrial cancer. Elevated preoperative CA-125 is associated with advanced FIGO stage, deep myometrial invasion, and lymph node and adnexal involvement, and is independently associated with disease-free and disease-specific survival, including among patients who would otherwise be classified as low risk [36,37]. In the present matched cohort, however, CA-125 did not achieve adequate balance after matching and was therefore removed from Table 1, which now presents the other baseline characteristics available in the TriNetX platform (demographics, sites of metastatic disease, recorded biomarkers, prior procedures, systemic therapy, and laboratory values); no statement about the contribution of CA-125 to the prognostic effect of the TFI can be made from our data, and this remains a question for future studies with complete tumor-marker ascertainment.

This study has several limitations. Its retrospective design introduces potential selection bias; although propensity score matching was applied, unmeasured confounding may persist. We regret that, because of the limitations of the TriNetX data source, several strong prognostic factors—FIGO stage, metastatic burden, histologic subtype, molecular subtype, and performance status—could not be retrieved as structured variables and were therefore not available for propensity score matching; the 77-covariate model used here represents the most complete adjustment the platform permits. Baseline hemoglobin and hematocrit remained lower in Cohort 1 after matching, which may contribute to the excess of grade 3 anemia. Baseline CA-125 was not adequately balanced after matching and was excluded from Table 1, so residual imbalance in this prognostic marker cannot be excluded. Data on key molecular markers—microsatellite instability, tumor mutational burden, programmed death-ligand 1 expression, and *POLE* status—were unavailable, and these could further explain heterogeneity in treatment response. Information on subsequent therapies after progression on pembrolizumab plus lenvatinib was limited, which may affect the interpretation of OS. Quality-of-life measures and patient-reported outcomes, which are particularly relevant in the recurrent or metastatic setting, were not captured. Treatment patterns and supportive care may vary across participating healthcare organizations, introducing institutional heterogeneity. Three of the 20 pre-specified outcomes could not be reported because patient counts fell below the data-network reporting threshold, and cell counts of 10 or fewer are censored in Table 1, so apparent balance in those rows cannot be confirmed. The proportional hazards assumption was violated for the two mortality endpoints, so the reported hazard ratios represent time-averaged effects, and a restricted mean survival time or piecewise analysis would complement these estimates. Defining cohorts by an interval that is only known once second-line treatment begins raises the possibility of selection and immortal-time bias, because patients in the longer-TFI cohort necessarily survived, and remained fit enough to be treated, until that interval had elapsed. Immortal-time bias in the survival comparison itself is avoided by the design, since follow-up began at the index date (first pembrolizumab plus lenvatinib administration) in both cohorts and the pre-index interval was not counted as follow-up (Section 2.5). Selection bias cannot be excluded, however: the longer-TFI cohort is by construction a population that reached second-line therapy in a condition to receive it, and the findings should be interpreted as conditional on receiving pembrolizumab plus lenvatinib. We also acknowledge that the TriNetX platform records only the dates of the two treatments; it provides neither a reliable progression date nor any measure of treatment eligibility such as performance status, organ function, or physician judgment. The TFI therefore conflates several distinct mechanisms—deferral of second-line therapy because disease remained controlled, recovery from chemotherapy toxicity, and referral or reimbursement delays—that cannot be separated in these data. A landmark analysis, or a Cox model in which treatment timing is entered as a time-varying covariate, would require patient-level data; like the restricted cubic spline and the continuous calendar-year adjustment discussed above, this is precluded by the aggregate-only output of the platform and would require a formal TriNetX data request, which was beyond the scope of this study; as an alternative sensitivity analysis, repeating the entire procedure with the TFI dichotomized at 3, 9, and 12 months gave associations of consistent direction, with the strongest mortality signal at 6 months (Table 4). Because all patients received pembrolizumab plus lenvatinib and no comparator regimen was analyzed, the study can establish only a prognostic association; it cannot determine whether the TFI modifies the treatment effect of the regimen, and no inference about the optimal timing of pembrolizumab plus lenvatinib should be drawn. Because patients with an adverse-event condition recorded before the index date were retained in the main analysis, the reported cumulative risks reflect the burden of each condition observed during follow-up in each cohort and not the incidence of treatment-emergent events; baseline prevalence was substantial for several conditions (Table 5). The sensitivity analysis restricted to treatment-emergent events reproduced the point estimates of the main analysis with wider confidence intervals, but seven outcomes became unreportable, and the matched structure was lost, so the adverse-event findings should be interpreted as a comparison between cohorts rather than as the toxicity to be expected from the regimen. Twenty outcomes were evaluated and, owing to the aggregate-only output of the TriNetX platform, no adjustment for multiple comparisons could be performed; we acknowledge that this increases the risk of type I error for the exploratory adverse-event analyses, and the associations with grade 3 anemia and hypothyroidism should be regarded as hypothesis-generating until confirmed in an independent dataset. The primary outcome of overall survival was a single prespecified comparison and is not affected by this concern. The binary 6-month TFI cut-off simplifies what is likely a continuous relationship. Modeling the TFI as a continuous variable, for example, with a restricted cubic spline, would be the preferred approach to determine whether the association with OS is linear or whether a clinically meaningful threshold exists near 6 months; however, such an analysis requires patient-level data (one record per patient with the TFI in days, follow-up time, vital status, and covariates), whereas the TriNetX Advanced Analytics platform returns only aggregate, cohort-level statistics (hazard ratios, Kaplan–Meier curve points, and risk tables) and does not export individual records, so a spline model could not be fitted. The 6-month threshold was chosen a priori because it is the cut-off conventionally used to define platinum sensitivity in gynecologic cancers [14,15,31,32]; whether a different threshold would be more informative in this setting remains to be tested in a dataset with patient-level access. In addition, the study period (2018–2026) spans a rapidly evolving treatment landscape, so treatment year is a potential confounder [38,39,40]. We were unable to adjust for calendar year or to stratify the analysis by treatment era, because the TriNetX platform does not release the number of patients treated in each year and does not permit calendar year to be used as a matching or stratification variable. We note, however, that both cohorts were assembled from the same 2018–2026 accrual window under identical eligibility criteria, and both include patients who survived into the period in which newer agents such as the HER2-directed antibody–drug conjugate trastuzumab deruxtecan became available; the influence of such subsequent therapies on overall survival is therefore expected to be similar in the two cohorts, although this could not be verified directly. Finally, the source population overlaps with that of our previous TriNetX analysis comparing pembrolizumab–lenvatinib with doxorubicin in patients with short treatment-free intervals [22]; the present study addresses a distinct question and a different comparison, but the overlap should be considered when interpreting the findings.

Despite these limitations, our results provide real-world evidence for the prognostic significance of the TFI in recurrent EC treated with pembrolizumab plus lenvatinib. The TFI can therefore be used as a reference for the survival outcome of patients with advanced or recurrent EC who receive this second-line regimen and for the comparison of adverse-event profiles between TFI groups.

## 5. Conclusions

A TFI ≥ 6 months after first-line carboplatin–paclitaxel is associated with significantly improved survival among patients with advanced or recurrent EC receiving second-line pembrolizumab plus lenvatinib, with a broadly similar toxicity profile. This association is prognostic: because all patients received the same regimen, the findings do not imply that the timing of pembrolizumab plus lenvatinib alters its effect or that deferring treatment improves survival. The TFI can therefore serve as a real-world evidence-based reference for the prognosis of patients with advanced or recurrent EC treated with second-line pembrolizumab plus lenvatinib and for the comparison of adverse-event profiles between TFI groups.

## Figures and Tables

**Figure 1:**
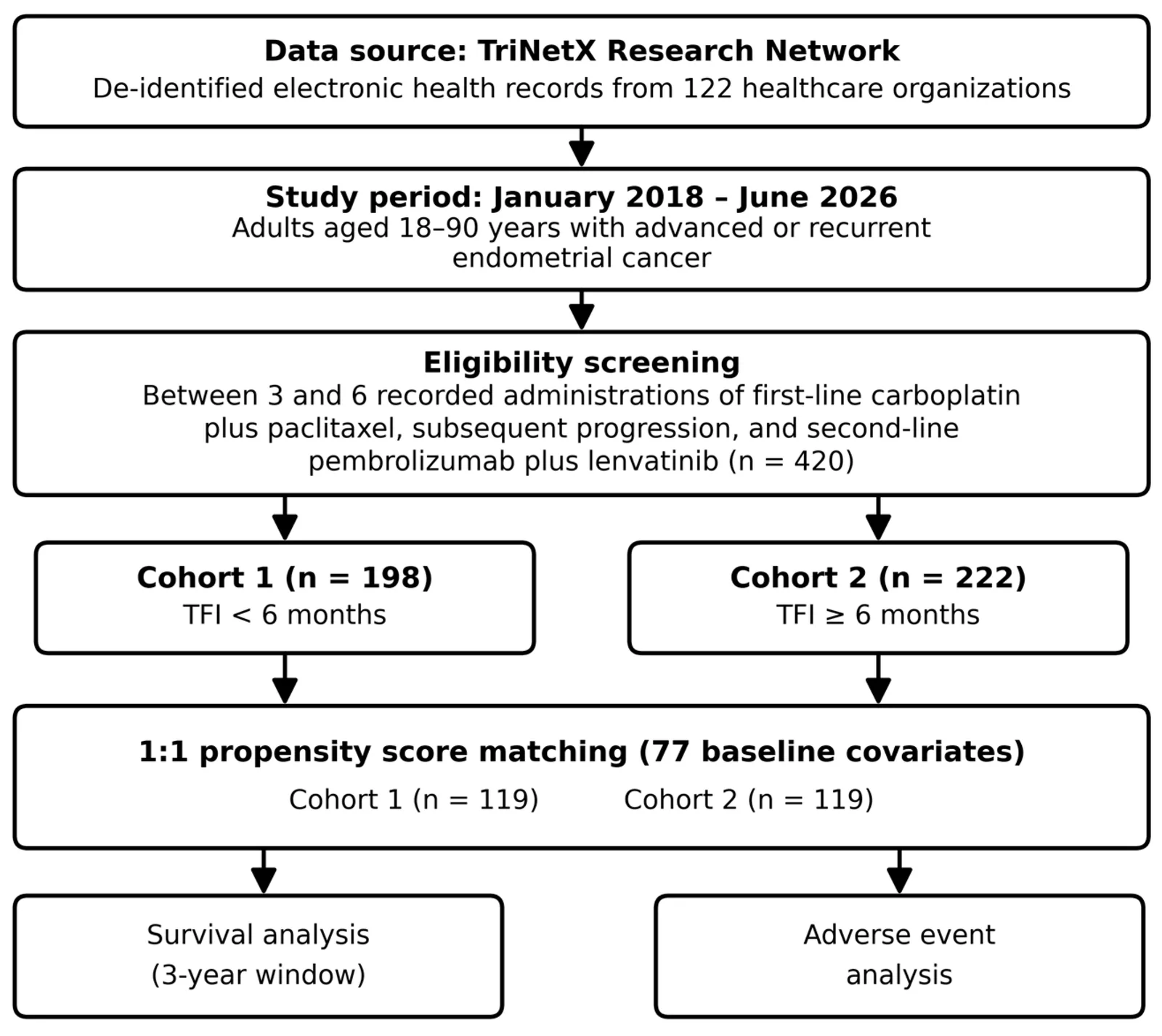
Study flow diagram. Patients were identified from the TriNetX Research Network, a federated database of de-identified electronic health records contributed by 122 healthcare organizations. Adults aged 18–90 years with advanced or recurrent endometrial cancer during January 2018 to June 2026 were screened for eligibility. Patients were eligible if they had between 3 and 6 recorded administrations of first-line carboplatin plus paclitaxel, documented subsequent progression, and receipt of second-line pembrolizumab plus lenvatinib (*n* = 420). Eligible patients were stratified by TFI into Cohort 1 (TFI < 6 months; *n* = 198) and Cohort 2 (TFI ≥ 6 months; *n* = 222). After 1:1 propensity score matching on 77 baseline covariates, 119 patients per cohort were retained and entered the survival analysis (3-year window) and the adverse-event analysis. TFI, treatment-free interval.

**Figure 2:**
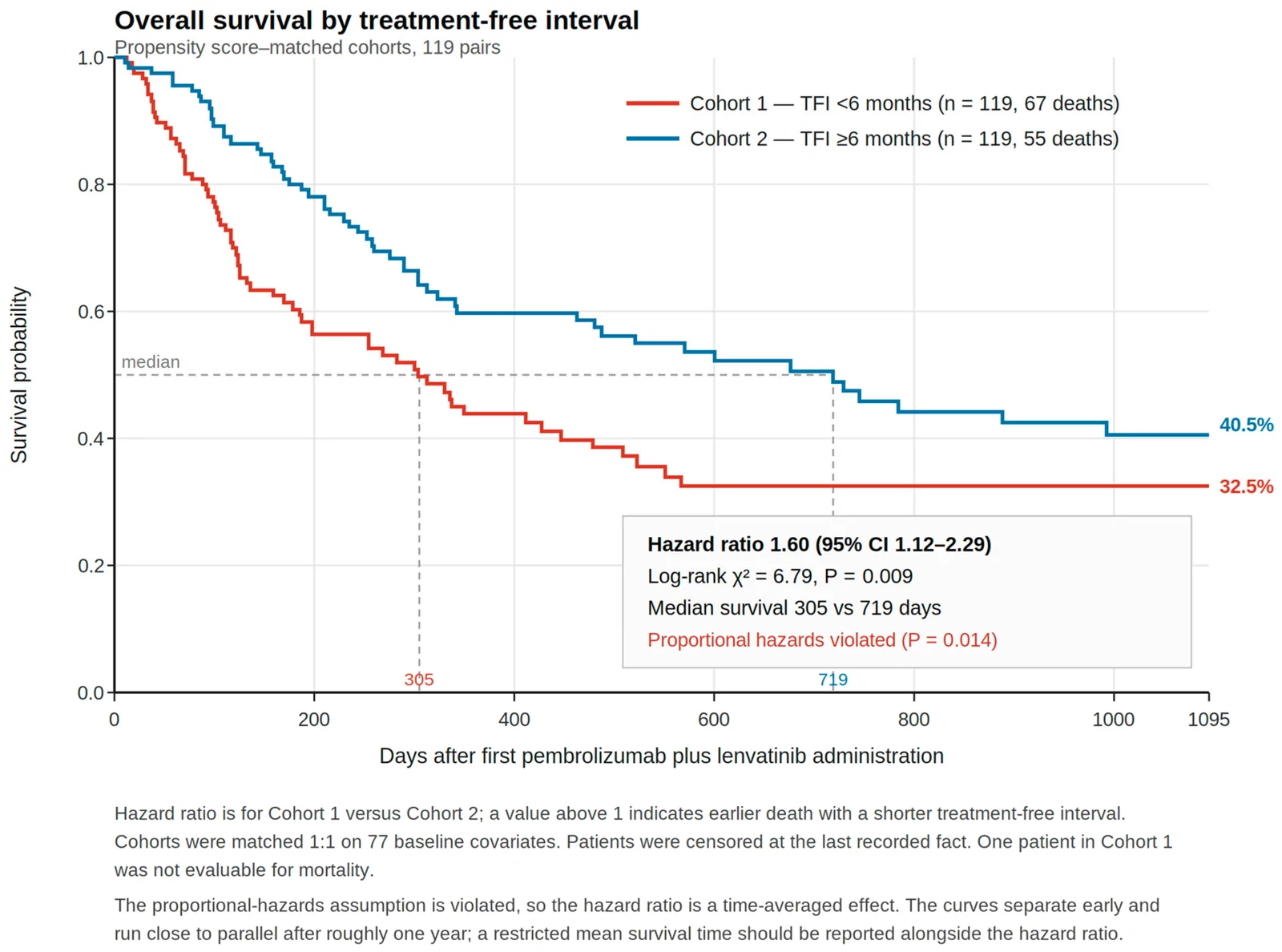
Kaplan–Meier estimates of overall survival by treatment-free interval. Overall survival is shown from the index event for the 1:1 propensity score-matched cohorts (*n* = 119 per group). Cohort 1 (red line) comprised patients with a TFI < 6 months (67 deaths) and Cohort 2 (blue line) those with a TFI ≥ 6 months (55 deaths); all patients received second-line pembrolizumab plus lenvatinib. Median overall survival was 305 days in Cohort 1 and 719 days in Cohort 2 (dashed vertical lines; the horizontal dashed line denotes 50% survival probability), and survival probability at 1095 days was 32.5% and 40.5%, respectively. The hazard ratio was 1.60 (95% CI 1.12–2.29) and the log-rank *p* value was 0.009. The proportional-hazards assumption was violated (*p* = 0.014), so the hazard ratio is a time-averaged effect. One patient in Cohort 1 was not evaluable for mortality. Patients were censored at the date of the last recorded fact in their electronic health record. TFI, treatment-free interval; CI, confidence interval.

**Figure 3:**
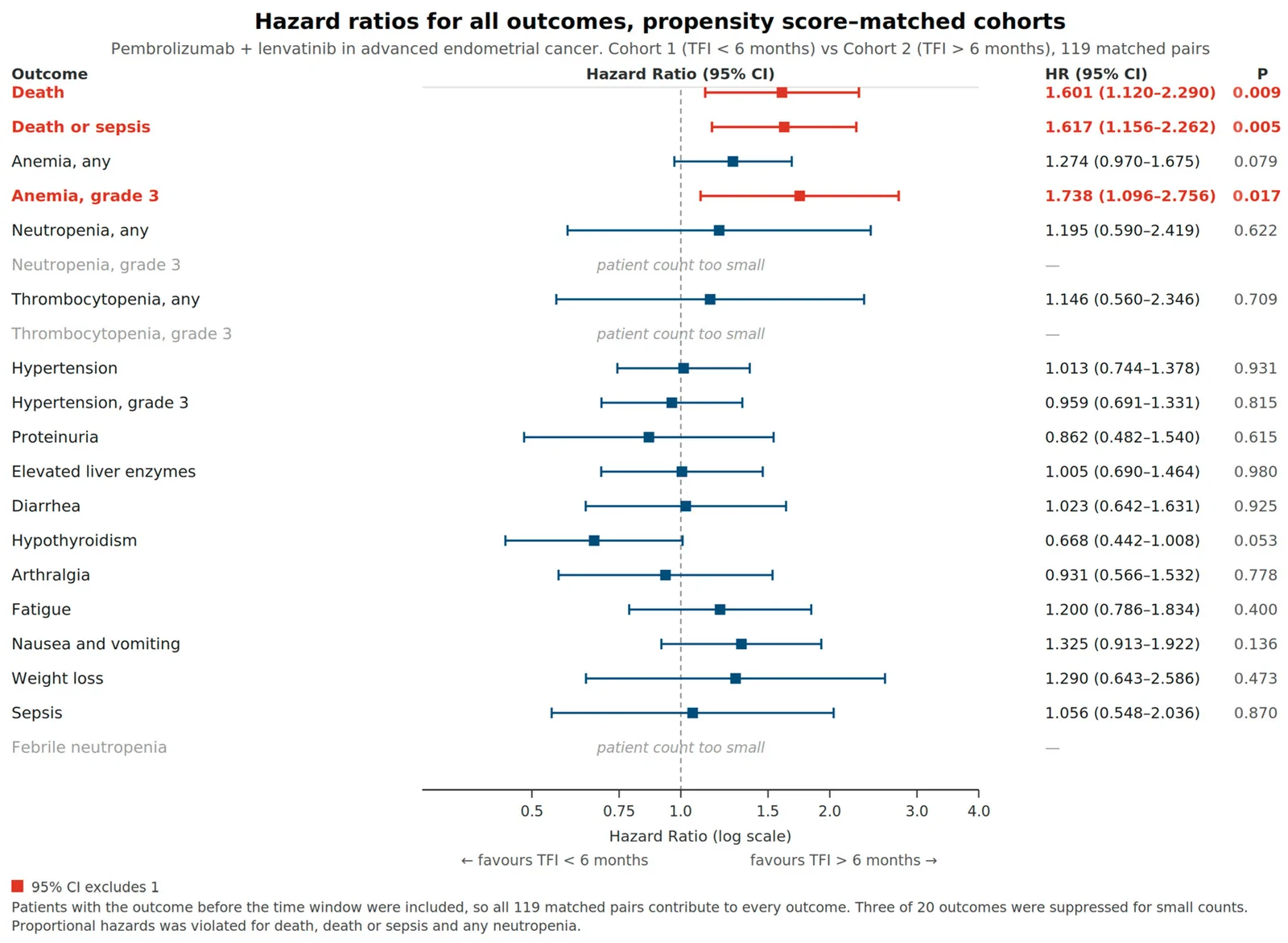
Forest plot of hazard ratios for the 20 pre-specified outcomes. Hazard ratios with 95% confidence intervals are shown on a logarithmic scale. HR > 1 indicates a higher event hazard in Cohort 1 (TFI < 6 months) versus Cohort 2 (TFI ≥ 6 months). Red denotes a 95% CI excluding 1. Three outcomes (grade 3 neutropenia, grade 3 thrombocytopenia, and febrile neutropenia) were suppressed because the patient count fell below the data-network reporting threshold. CI, confidence interval; TFI, treatment-free interval.

**Table 4:** Sensitivity analysis: hazard ratios (Cohort 1 versus Cohort 2) for alternative treatment-free interval thresholds.

Outcome	TFI 3 Months	TFI 6 Months (Primary)	TFI 9 Months	TFI 12 Months
**Cohort size**				
Cohort 1/Cohort 2 before matching, *n*	98/323	198/222	270/151	321/104
Matched pairs, *n*	77	119	94	65
**Hazard ratio (** ***p*** **value)**				
Death	1.443 (0.096)	**1.601 (0.009)**	1.440 (0.084)	1.479 (0.097)
Death or sepsis	**1.529 (0.044)**	**1.617 (0.005)**	1.444 (0.069)	1.553 (0.053)
Anemia, any	**1.735 (0.0015)**	1.274 (0.079)	**1.559 (0.029)**	**1.519 (0.030)**
Anemia, grade 3	**2.679 (0.0011)**	**1.738 (0.017)**	**1.989 (0.017)**	**3.014 (0.0009)**
Hypothyroidism	**0.566 (0.033)**	0.668 (0.053)	0.793 (0.296)	0.924 (0.763)

TFI, treatment-free interval. Cohort 1 = TFI shorter than the threshold; Cohort 2 = TFI equal to or longer than the threshold. For each threshold, the cohorts were rebuilt with identical eligibility criteria, matched 1:1 by propensity score on the same 77 baseline covariates, and analyzed by the Kaplan–Meier method from the same index date (first administration of pembrolizumab plus lenvatinib) over 1095 days. Hazard ratios are for Cohort 1 versus Cohort 2; *p* values are from the log-rank test. Bold denotes *p* < 0.05. The 12-month analysis is based on the smallest matched sample (65 pairs) and should be interpreted with caution.

**Table 5:** Baseline prevalence of each adverse-event condition and sensitivity analysis restricted to treatment-emergent events (patients with the condition recorded before the index date excluded).

Outcome	Baseline Prevalence, *n* (%)		Patients at Risk		Events		HR (95% CI), Treatment-Emergent	Log-Rank *p*	HR, Main Analysis (Table 3)
	C1	C2	C1	C2	C1	C2			
**Mortality**									
Death ^a^	—	—	118	119	67	55	**1.601 (1.120–2.290)**	**0.009**	1.601
Death or sepsis ^b^	17 (14.3)	4 (3.4)	102	115	63	62	**1.508 (1.060–2.145)**	**0.021**	1.617
**Hematologic toxicity**									
Anemia, any	*Suppressed—patient count below the data-network reporting threshold after exclusion*								
Anemia, grade 3	56 (47.1)	38 (31.9)	63	81	16	15	1.744 (0.859–3.541)	0.119	1.738
Neutropenia, any	*Suppressed—patient count below the data-network reporting threshold after exclusion*								
Neutropenia, grade 3	*Suppressed—patient count below the data-network reporting threshold after exclusion*								
Thrombocytopenia, any	*Suppressed—patient count below the data-network reporting threshold after exclusion*								
Thrombocytopenia, grade 3	*Suppressed—patient count below the data-network reporting threshold after exclusion*								
**Lenvatinib-associated toxicity**									
Hypertension	83 (69.7)	82 (68.9)	36	37	12	12	1.017 (0.457–2.266)	0.967	1.013
Hypertension, grade 3 ^c^	84 (70.6)	90 (75.6)	35	29	13	17	0.647 (0.313–1.337)	0.235	0.959
Proteinuria	*Suppressed—patient count below the data-network reporting threshold after exclusion*								
Elevated liver enzymes	37 (31.1)	40 (33.6)	82	79	28	33	0.870 (0.525–1.440)	0.585	1.005
Diarrhea	28 (23.5)	24 (20.2)	91	95	24	26	1.123 (0.644–1.959)	0.684	1.023
**Immune-related and constitutional**									
Hypothyroidism	17 (14.3)	24 (20.2)	102	95	27	35	0.811 (0.490–1.340)	0.411	0.668
Arthralgia	43 (36.1)	47 (39.5)	76	72	16	16	1.132 (0.565–2.268)	0.725	0.931
Fatigue	42 (35.3)	42 (35.3)	77	77	16	19	0.898 (0.461–1.748)	0.751	1.200
Nausea and vomiting	49 (41.2)	55 (46.2)	70	64	27	19	1.366 (0.759–2.458)	0.296	1.325
Weight loss	6 (5.0)	11 (9.2)	113	108	14	13	1.183 (0.555–2.521)	0.662	1.290
**Infection**									
Sepsis	*Suppressed—patient count below the data-network reporting threshold after exclusion*								
Febrile neutropenia	*Suppressed—patient count below the data-network reporting threshold after exclusion*								

C1, Cohort 1 (TFI < 6 months); C2, Cohort 2 (TFI ≥ 6 months); CI, confidence interval; HR, hazard ratio. Baseline prevalence is the number of patients with the condition recorded before day 1 after the index date, derived as 119 minus the number of patients at risk after exclusion; percentages use 119 as the denominator. For each outcome, patients with that outcome before the index date were excluded, so denominators differ by outcome and the matched pairs are no longer intact. Hazard ratios are for Cohort 1 versus Cohort 2 over days 1 to 1095. Bold denotes a 95% CI excluding the null or *p* < 0.05. ^a^ One patient in Cohort 1 was not evaluable for mortality; death has no baseline prevalence. ^b^ Exclusion based on sepsis recorded before the index date. ^c^ The median event-free times for grade 3 hypertension (966 versus 203 days) with identical event-free probability at 1095 days reflect the small numbers at risk and should not be interpreted. The proportional-hazards assumption was violated for death (*p* = 0.014) but not for death or sepsis (*p* = 0.110) in this analysis.

## Data Availability

The data underlying this study were obtained from the TriNetX Research Network under a license agreement and are not publicly available. Access to the TriNetX platform may be requested from TriNetX LLC (https://trinetx.com). The aggregated data supporting the findings reported here are available from the corresponding authors upon reasonable request.
